# Prognostic significance of inflammatory biomarkers in hepatocellular carcinoma following hepatic resection

**DOI:** 10.1002/bjs5.50170

**Published:** 2019-04-29

**Authors:** S. Itoh, K. Yugawa, M. Shimokawa, S. Yoshiya, Y. Mano, K. Takeishi, T. Toshima, Y. Maehara, M. Mori, T. Yoshizumi

**Affiliations:** ^1^ Department of Surgery and Science, Graduate School of Medical Sciences Kyushu University Fukuoka Japan; ^2^ Department of Surgery Kyushu Central Hospital of the Mutual Aid Association of Public School Teachers Fukuoka Japan

## Abstract

**Background:**

Cancer‐related inflammation has been correlated with cancer prognosis. This study evaluated inflammatory biomarkers, including neutrophil‐to‐lymphocyte ratio (NLR), platelet‐to‐lymphocyte ratio (PLR) and lymphocyte‐to‐monocyte ratio (LMR), programmed death ligand (PD‐L) 1 expression, and tumour microenvironment in relation to prognosis and clinicopathological features of patients with hepatocellular carcinoma (HCC) undergoing curative hepatic resection.

**Methods:**

Patients who had liver resection for HCC in 2000–2011 were analysed. Univariable and multivariable analyses were conducted for overall (OS) and recurrence‐free (RFS) survival. Immunohistochemical analyses of PD‐L1, CD8 and CD68 expression were performed. HCC cell lines were evaluated for PD‐L1 expression. A subgroup analysis was conducted to determine patient features, survival and the tumour microenvironment. Results were validated in a cohort of patients with HCC treated surgically in 2012–2016.

**Results:**

Some 281 patients who underwent hepatic resection for HCC were included. Multivariable analysis showed that low LMR was an independent prognostic factor of OS (hazard ratio (HR) 1·59, 95 per cent c.i. 1·00 to 2·41; *P* = 0·045) and RFS (HR 1·47, 1·05 to 2·04; *P* = 0·022) after resection. Low preoperative LMR values were correlated with higher α‐fetoprotein values (*P* < 0·001), larger tumour size (*P* < 0·001), and high rates of poor differentiation (*P* = 0·035) and liver cirrhosis (*P* = 0·008). LMR was significantly lower in PD‐L1‐positive patients than in those with PD‐L1 negativity (*P* < 0·001). Results were confirmed in the validation cohort. PD‐L1 expression was upregulated in HCC cell lines treated with interferon‐γ and co‐cultured with THP‐1 monocyte cells.

**Conclusion:**

LMR is an independent predictor of survival after hepatic resection in patients with HCC. Modulation of the immune checkpoint pathway in the tumour microenvironment is associated with a low LMR.

## Introduction

Hepatocellular carcinoma (HCC) is the fifth most common cancer worldwide and is considered a major cause of cancer‐related death^1^. Hepatic resection is a safe and effective treatment in patients with HCC and well preserved liver function. However, the proportion of patients who develop intrahepatic recurrence remains high^2^, ^3^.

In recent decades a number of studies have focused on inflammation, highlighting that cancer‐related inflammation plays an important role in the development of cancer. The host response in systemic inflammation is associated with disturbance of various haematological components, such as white blood cells (specifically neutrophils, lymphocytes and monocytes) and platelets, and has been considered an independent prognostic factor in various types of cancer^4^. In HCC, the pretreatment peripheral neutrophil‐to‐lymphocyte ratio (NLR) has been reported^5^ to be an independent predictor of survival. The platelet‐to‐lymphocyte ratio (PLR) has also been correlated with prognosis of HCC^6^, and the lymphocyte‐to‐monocyte ratio (LMR) has been investigated recently as a prognostic marker in patients with solid tumours^7,8^, including patients with HCC who underwent hepatic resection^9^, ^10^.

A recent study^11^ showed that inhibition of the programmed death (PD) 1/programmed death ligand (PD‐L) 1 immune checkpoint pathway improved the survival of patients with melanoma and lung cancer. PD‐1 is expressed on the surface of T cells and manipulates their activity by interaction with its ligands, PD‐L1 and PD‐L2. The interaction between PD‐1 and PD‐L1 or PD‐L2 attenuates T‐cell activity, resulting in downregulation of the immune response against cancer cells. Inhibition of these interactions using PD‐1‐ or PD‐L1‐blocking antibodies suppresses cancer‐cell immune escape and induces a T cell‐mediated immune response to the cancer cells^12^.

In the present study, inflammatory biomarkers, including NLR, PLR and LMR, and the tumour microenvironment (PD‐L1 expression, intratumour lymphocytes and macrophages) were investigated in relation to the clinical features and prognosis of patients who had surgical resection for HCC.

## Methods

All consecutive patients who underwent hepatic resection for HCC as a first treatment from January 2000 to December 2011 at the Department of Surgery and Science, Kyushu University Hospital were reviewed. Exclusion criteria for data analysis were a previous splenectomy and/or a platelet count of 100 000/mm^3^ or above.

The study was approved by the ethics committee of Kyushu University (approval codes: 28‐289 and 28‐453).

NLR, PLR and LMR were calculated based on preoperative blood values. The white blood count was measured 1 day before hepatic resection.

Data retrieved and analysed included age, sex, BMI, hepatitis B surface antigen, hepatitis C virus antibody, diabetes mellitus, Child–Pugh classification, oesophageal varix, total bilirubin, albumin, prothrombin time, indocyanine green retention rate at 15 min (ICGR15), α‐fetoprotein (AFP), *des*‐γ‐carboxyprothrombin (DCP), tumour size, number of lesions, differentiation, microvascular invasion, microscopic intrahepatic metastasis, liver cirrhosis, duration of surgery, blood loss and blood transfusion.

### Surgical procedures

Surgical techniques and patient selection criteria for resection of HCC have been reported previously^13^. Selection criteria for hepatic resection were: ascites not detected or controllable by diuretics; serum total bilirubin level below 2·0 mg/ml; and ICGR15 less than 40 per cent. Parenchymal transection was performed using the cavitron ultrasonic surgical aspirator (CUSA®; Valleylab, Boulder, Colorado, USA). Inflow vascular control was performed with intermittent hemiocclusion or total occlusion of Glisson's sheath.

### Follow‐up

Patients had monthly AFP and DCP measurements as well as monthly ultrasonography performed in outpatients, from hospital discharge to death. Dynamic CT was performed by radiologists every 3 months, and angiographic examination was done upon admission if recurrence was suspected. Overall survival (OS) was defined as death from any cause. Recurrence‐free survival (RFS) was defined as first recurrence after resection.

### Immunohistochemical examination

Sections of resected specimens were fixed in 10 per cent buffered formalin, embedded in paraffin, and stained using the peroxidase‐labelled streptavidin–biotin technique with the Histofine® SAB‐PO kit (Nichirei Biosciences, Tokyo, Japan)^14^. The primary antibody used was an antihuman PD‐L1 rabbit monoclonal antibody (clone 28‐8, dilution 1 : 450; Abcam, Cambridge, UK). Tumour cells showed membranous staining for PD‐L1, which was evaluated as positive staining. The proportion of PD‐L1‐positive tumour cells was estimated as the percentage of total tumour cells. Because the distribution of PD‐L1‐positive cells was focal, positivity for PD‐L1 was defined as more than 1 per cent of tumour cells stained for PD‐L1^15^. Sections from human placenta were used as positive controls. Immunohistochemical (IHC) staining was performed using anti‐CD8 (C8/144B; DAKO, Carpinteria, California, USA) and anti‐CD68 (PG‐M1, DAKO) antibodies. Serial sections were treated before incubation with primary antibodies in a microwave oven for 20 min, reacted with the antibodies. The numbers of cells with cytoplasm or membrane staining in five high‐power fields (HPFs) were counted. The CD8+/CD68+ cell ratio was calculated from the median numbers of CD8+ and CD68+ cells.

### Cell culture

Human HCC cell lines, Huh7 and Hep3B (Riken Cell Bank, Tsukuba, Japan), were cultured as described previously^14^, ^16^. The human monocyte cell line THP‐1 (obtained from Culture Collections of Public Health England) was grown on RPMI‐1640 medium (Wako, Osaka, Japan) containing 10 per cent fetal bovine serum and 10 mmol HEPES. To mimic the interaction of cancer cells and macrophages in the tumour microenvironment, Huh7 and Hep3B cells were co‐cultured with THP‐1 cells for 48 h and harvested for extraction of proteins after removal of THP‐1 cells, according to the authors' protocol. To induce PD‐L1 expression on cancer cells, Huh7 and Hep3B cells were treated with 100 ng/ml recombinant interferon‐γ (IFN‐γ) (R&D Systems, Minneapolis, Minnesota, USA) for 48 h, and harvested for protein extraction.

### Western blotting

Western blotting was performed as described previously^14^, ^16^. The primary antibodies for PD‐L1 (clone 28‐8, dilution 1 : 450; Abcam) and β‐actin (clone AC‐15; Sigma‐Aldrich, St Louis, Missouri, USA) were used following the manufacturers' protocol.

### Statistical analysis

Data are expressed as median (range) values. Continuous variables without a normal distribution and variables such as the data using cell lines were compared with the Mann–Whitney *U* test. Categorical variables were compared with the χ^2^ or Fisher's exact test. Survival data were used to establish a univariable Cox proportional hazards model. Co‐variables that were significant at *P* < 0·050 were included in the multivariable Cox proportional hazards model. OS and RFS rates were calculated by the Kaplan–Meier method and compared with the rank test. Differences were considered significant at *P* < 0·050.

For analysis of NLR, PLR and LMR, cutoff values were determined by the receiver operating characteristic (ROC) curve with the endpoint of OS.

A subgroup analysis was conducted with the endpoint of detailing patient features, survival and tumour microenvironment. Specifically, patients were categorized according to inflammatory biomarker expression on the basis of cutoff values, and then clinical/pathological features, tumour microenvironment, IHC expression and survival were compared.

A validation cohort of patients treated between 2012 and 2016 with the same indications, and selected using the same inclusion and exclusion criteria, was used to confirm the results of the developing cohort study analysis.

All statistical analyses were performed using JMP® software (SAS Institute, Cary, North Carolina, USA).

## Results

Of 367 patients who underwent liver resection for HCC during the study period (2000–2011), 281 (224 men and 57 women) met the inclusion criteria. Their median age was 68 (range 28–87) years. Median follow‐up was 5·7 (range 0·1–15·8) years.

A total of 176 patients (62·6 per cent) presented with recurrent HCC (150 intrahepatic and 26 extrahepatic). Treatment for intrahepatic recurrence included hepatic resection (47 patients), local ablation therapy (27), transarterial chemoembolization (68) and hepatic arterial infusion chemotherapy (8). The best cutoff points for NLR, PLR and LMR were 1·42, 129·5 and 4·28 respectively, although the area under the ROC curve (AUC) was of limited value (0·568, 0·570 and 0·616 respectively) (*Fig*. S1, supporting information).

### Survival

Results of the univariable and multivariable analyses used to identify the factors significantly associated with OS after hepatic resection in patients with HCC are shown in *Table* 1. Univariable analysis showed that the poor prognostic factors for OS were older age, tumour size, macroscopic multiple tumours, poor differentiation, microvascular invasion, microscopic intrahepatic metastases, AFP level, DCP level, high NLR and PLR, and low LMR. Multivariable analysis showed that age, microscopic intrahepatic metastases, AFP concentration and low LMR (hazard ratio (HR) 1·59, 95 per cent c.i. 1·00 to 2·41; *P* = 0·045) were poor prognostic factors for OS.

**Table 1:** Cox proportional hazards analysis of factors related to overall survival in patients with hepatocellular carcinoma who underwent hepatic resection

	Univariable analysis		Multivariable analysis	
	Hazard ratio	*P*	Hazard ratio	*P*
Age (years)	1·03 (1·00, 1·05)	0·005	1·03 (1·00, 1·05)	0·005
Male sex	1·10 (0·69, 1·83)	0·695		
HBsAg+	0·81 (0·47, 1·29)	0·395		
HCV‐Ab+	1·14 (0·78, 1·68)	0·484		
Child–Pugh grade B	1·07 (0·23, 18·80)	0·944		
Liver cirrhosis	1·06 (0·65, 1·65)	0·785		
Tumour size	5·50 (2·37, 12·10)	< 0·001	1·44 (0·39, 4·80)	0·579
Macroscopic multiple tumours	2·47 (1·59, 3·73)	< 0·001	1·45 (0·82, 2·39)	0·197
Poor differentiation	1·95 (1·33, 2·84)	< 0·001	1·50 (0·74, 1·81)	0·487
Microvascular invasion	1·92 (1·31, 2·79)	< 0·001	1·52 (0·92, 2·41)	0·063
Microscopic intrahepatic metastases	3·30 (2·08, 5·07)	< 0·001	2·46 (1·40, 4·29)	0·001
AFP level	64·2 (8·99, 286·00)	< 0·001	21·6 (1·56, 300·00)	0·021
DCP level	2·56 (1·06, 5·32)	0·036	0·51 (0·13, 1·97)	0·332
High NLR	1·74 (1·15, 2·71)	0·007	1·24 (0·75, 2·07)	0·393
High PLR	1·71 (1·14, 2·81)	0·009	0·88 (0·54, 1·43)	0·630
Low LMR	1·93 (1·32, 2·81)	< 0·001	1·59 (1·00, 2·41)	0·045

Values in parentheses are 95 per cent confidence intervals. HBsAg, hepatitis B surface antigen; HCV‐Ab, hepatitis C virus antibody; AFP, α‐fetoprotein; DCP, *des*‐γ‐carboxyprothrombin; NLR, neutrophil‐to‐lymphocyte ratio; PLR, platelet‐to‐lymphocyte ratio; LMR, lymphocyte‐to‐monocyte ratio.

Univariable analysis also showed that liver cirrhosis, tumour size, macroscopic multiple tumours, poor differentiation, microvascular invasion, microscopic intrahepatic metastases, AFP and DCP concentration, and low LMR were significant predictors of RFS. Multivariable analysis found that liver cirrhosis, microscopic intrahepatic metastases, AFP concentration and low LMR (HR 1·47, 95 per cent c.i. 1·05 to 2·04; *P* = 0·022) remained as significant independent predictors of RFS (*Table* 2).

**Table 2:** Cox proportional hazards analysis of factors related to recurrence‐free survival in patients with hepatocellular carcinoma who underwent hepatic resection

	Univariable analysis		Multivariable analysis	
	Hazard ratio	*P*	Hazard ratio	*P*
Age (years)	0·99 (0·97, 1·00)	0·318		
Male sex	1·24 (0·85, 1·86)	0·260		
HBsAg+	0·83 (0·55, 1·20)	0·340		
HCV‐Ab+	1·31 (0·96, 1·78)	0·079		
Child–Pugh grade B	1·11 (0·35, 6·74)	0·875		
Liver cirrhosis	1·56 (1·10, 2·17)	0·012	1·62 (1·11, 2·34)	0·012
Tumour size	4·08 (1·91, 8·32)	< 0·001	1·48 (0·52, 3·93)	0·447
Macroscopic multiple tumours	2·38 (1·64, 3·37)	< 0·001	1·45 (0·91, 2·23)	0·108
Poor differentiation	1·56 (1·13, 2·13)	0·006	1·04 (0·70, 1·51)	0·838
Microvascular invasion	1·36 (1·00, 1·84)	0·049	1·01 (0·70, 1·47)	0·933
Microscopic intrahepatic metastases	3·88 (2·61, 5·62)	< 0·001	2·95 (1·83, 4·67)	< 0·001
AFP level	170·70 (24·50, 937·00)	< 0·001	27·30 (2·60, 190·00)	0·009
DCP level	6·49 (2·86, 13·00)	< 0·001	2·79 (0·92, 7·55)	0·068
High NLR	1·27 (0·93, 1·75)	0·130		
High PLR	1·16 (0·82, 1·62)	0·382		
Low LMR	1·80 (1·32, 2·43)	< 0·001	1·47 (1·05, 2·04)	0·022

Values in parentheses are 95 per cent confidence intervals. HBsAg, hepatitis B surface antigen; HCV‐Ab, hepatitis C virus antibody; AFP, α‐fetoprotein; DCP, *des*‐γ‐carboxyprothrombin; NLR, neutrophil‐to‐lymphocyte ratio; PLR, platelet‐to‐lymphocyte ratio; LMR, lymphocyte‐to‐monocyte ratio.

### Tumour microenvironment

**Figure 1:**
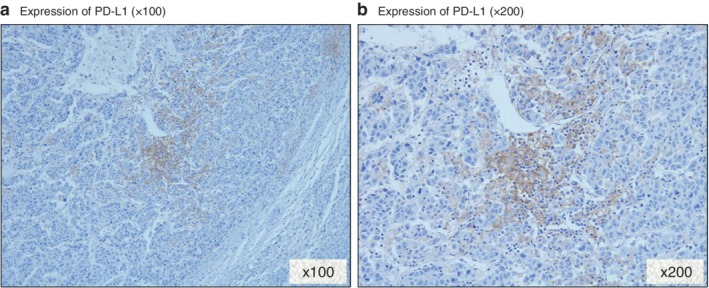
Immunohistochemical staining of programmed death ligand (PD‐L) 1 in a hepatocellular carcinoma specimen. Positive expression of PD‐L 1 in the membrane of hepatocellular carcinoma cells: **a** magnification ×100; **b** magnification ×200

Analysis of PD‐L1 was conducted in 223 patients who underwent hepatic resection for HCC, owing to a shortage of pathological sections (*Fig*. 1 *a,b*). Forty‐two patients (18·8 per cent) were PD‐L1+. Inflammatory biomarkers were compared in PD‐L1+ and PD‐L− patients.

**Figure 2:**
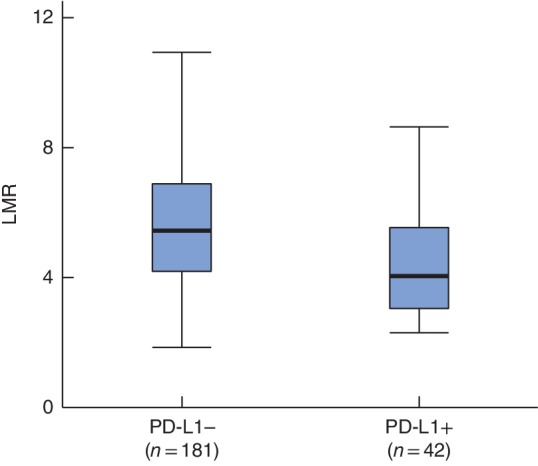
Box‐and‐whisker plot of lymphocyte‐to‐monocyte ratio in patients with programmed death ligand 1 positivity and negativity. Median values, interquartile ranges and ranges are denoted by horizontal bars, boxes and error bars respectively. LMR, lymphocyte‐to‐monocyte ratio; PD‐L1, programmed death ligand 1. *P* < 0·001 (Mann–Whitney *U* test)

**Figure 3:**
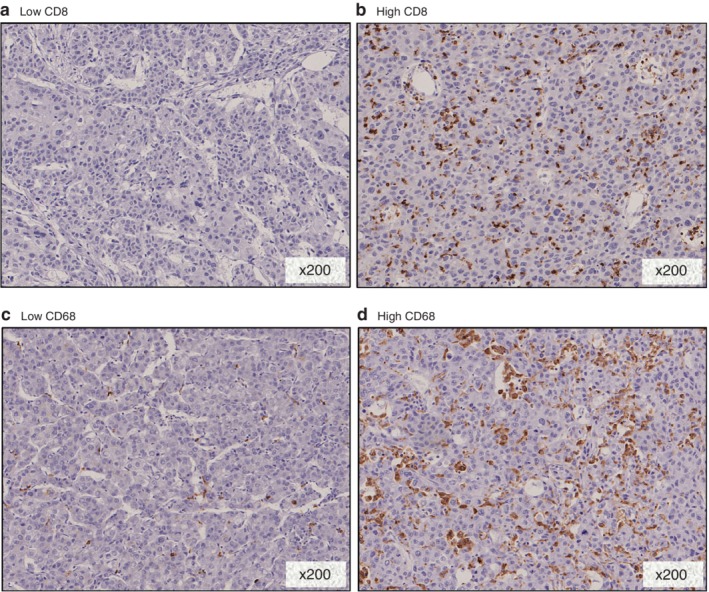
Immunohistochemical staining of CD8 and CD68 in a hepatocellular carcinoma (HCC) specimen. **a** Low and **b** high staining of CD8 in HCC specimens. **c** Low and **d** high staining of CD68 in HCC specimens. **a–d** Magnification ×200

LMR was significantly lower in PD‐L1+ than in PD‐L1− patients (median (range) 4·05 (2·27–8·66) *versus* 5·42 (1·87–10·94) respectively; *P* < 0·001) (*Fig*. 2). There was no statistically significant association between PD‐L1 expression and NLR (*P* = 0·073) or PLR (*P* = 0·051). CD8 and CD68 IHC expression was tested in 64 sections, owing to a shortage of pathological sections (*Fig*. 3).

### LMR and clinicopathological features

Patients were grouped according to LMR values, differentiating those with a high LMR (4·28 or above; 187 patients) from those with a low LMR (less than 4·28; 94 patients); their clinical and pathological characteristics are shown in *Table* 3. A higher LMR value was significantly correlated with a high serum albumin concentration (*P* < 0·001), high prothrombin time (*P* = 0·036) and less liver cirrhosis (*P* = 0·008). A lower preoperative LMR correlated with a high AFP concentration (*P* < 0·001), large tumour size (*P* < 0·001) and high rate of poorly differentiated HCC (*P* = 0·035).

**Table 3:** Characteristics of patients with hepatocellular carcinoma who underwent hepatic resection

	High LMR (*n* = 187)	Low LMR (*n* = 94)	*P*
Age (years)*	68 (31–87)	69 (28–85)	0·741
Sex ratio (M : F)	148 : 39	76 : 18	0·737
BMI (kg/m^2^)*	22·7 (15·7–32·1)	23·0 (14·2–30·9)	0·126
HBsAg+	38 (20·3)	16 (17)	0·507
HCV‐Ab+	105 (56·1)	58 (62)	0·373
Diabetes mellitus	55 (29·4)	26 (28)	0·759
Child–Pugh grade			0·553
A	184 (98·4)	94 (100)	
B	3 (1·6)	0 (0)	
Oesophageal varix	12 (6·4)	7 (7·4)	0·745
Total bilirubin (mg/dl)*	0·7 (0·3–2·6)	0·7 (0·2–1·5)	0·537
Albumin (g/dl)*	4·1 (2·8–5·3)	3·9 (1·8–4·8)	< 0·001
Prothrombin time (%)*	89 (63–116)	96 (65–116)	0·036
ICGR15 (%)*	13 (1·6–43·1)	11·5 (3·0–34·6)	0·454
AFP (ng/ml)*	7·6 (0·5–170 668)	18·2 (1·6–994 600)	< 0·001
DCP (munits/ml)*	73 (9–75 000)	111 (2–75 000)	0·115
Tumour size (cm)*	3·2 (0·9–13·0)	4·0 (1·2–16·5)	< 0·001
No. of tumours			
1	155 (82·9)	75 (80)	0·524
≥ 2	32 (17·1)	19 (20)	
Poor differentiation	47 (25·1)	35 (37)	0·035
Microvascular invasion	59 (31·6)	37 (39)	0·192
Microscopic intrahepatic metastases	27 (14·4)	16 (17)	0·570
Liver cirrhosis	31 (16·6)	29 (31)	0·008
Duration of surgery (min)*	325 (117–759)	321 (147–770)	0·389
Blood loss (ml)*	500 (0–13 000)	568 (5–5723)	0·229
Blood transfusion	16 (8·6)	13 (14)	0·170

Values in parentheses are percentages unless indicated otherwise;

* values are median (range). LMR, lymphocyte‐to‐monocyte ratio; HBsAg, hepatitis B surface antigen; HCV‐Ab, hepatitis C virus antibody; ICGR15, indocyanine green retention rate at 15 min; AFP, α‐fetoprotein; DCP, *des*‐γ‐carboxyprothrombin.

### LMR and the tumour microenvironment

The tumour immunogenic microenvironment was compared in patients with high and low LMR values. There was no statistically significant association between LMR and CD8+ cells (*P* = 0·328). However, the number of CD68+ cells was significantly higher in patients with a low LMR than in those with a high LMR (median (range) 101 (31–162) *versus* 87 (24–157) respectively; *P* = 0·020).

**Figure 4:**
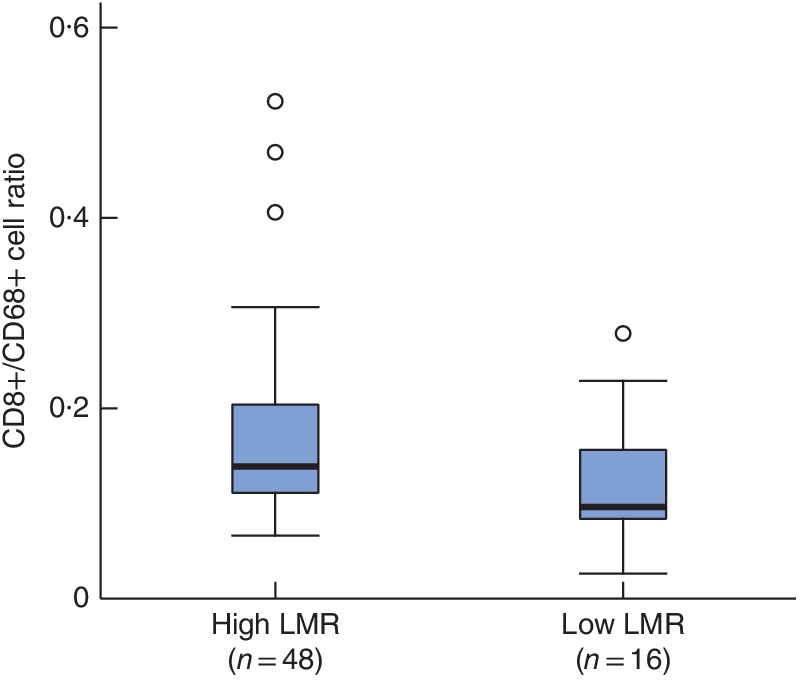
Box‐and‐whisker plot of CD8+/CD68+ cell ratios in patients with high and low lymphocyte‐to‐monocyte ratios. Median values, interquartile ranges and ranges (excluding outliers) are denoted by horizontal bars, boxes and error bars respectively. LMR, lymphocyte‐to‐monocyte ratio. *P* = 0·010 (Mann–Whitney *U* test)

The CD8+/CD68+ cell ratio was significantly lower in the low LMR group (median (range) 0·09 (0·02–0·23) *versus* 0·14 (0·06–0·30) respectively; *P* = 0·010) (*Fig*. 4).

### LMR and survival

**Figure 5:**
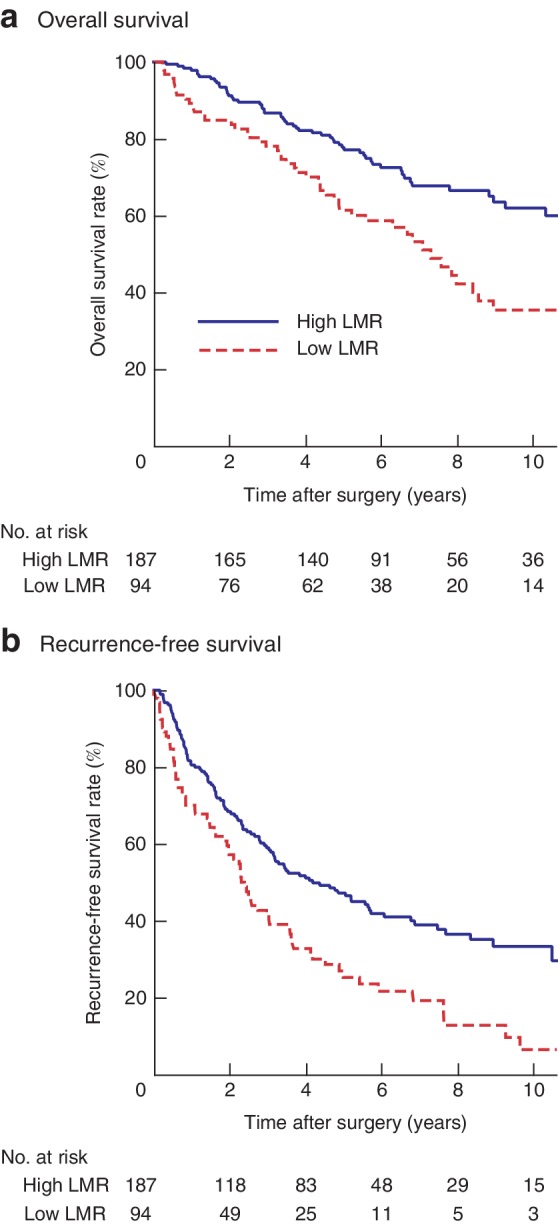
a,b Kaplan–Meier survival curves after resection of hepatocellular carcinoma in patients with high and low lymphocyte‐to‐monocyte ratios. **a** Overall and **b** recurrence‐free survival. LMR, lymphocyte‐to‐monocyte ratio. **a,b** *P* < 0·001 (log rank test)

The 5‐ and 10‐year OS rates in high and low LMR groups were 77·7 *versus* 61 per cent and 61·9 *versus* 35 per cent respectively (*P* < 0·001) (*Fig*. 5a). The 5‐ and 10‐year RFS rates in high and low LMR groups were 47·1 *versus* 25 per cent and 33·2 *versus* 6 per cent respectively (*P* < 0·001) (*Fig*. 5b).

Low LMR was significantly associated with poor OS and RFS in patients without liver cirrhosis, and with poor RFS in patients with cirrhosis (*Fig*. S2, supporting information). Low LMR was also significantly associated with worse survival in patients with and without microvascular invasion (*Fig*. S3, supporting information).

Patients were also divided into four groups: high LMR/PD‐L1− (134 patients); high LMR/PD‐L1+ (19); low LMR/PD‐L1− (47); low LMR/PD‐L1+ (23). Both OS (*P* < 0·001) and RFS (*P* = 0·007) rates were significantly improved in the high LMR/PD‐L1− group compared with rates in the other three groups (*Fig*. S4, supporting information).

### Validation cohort

Of 187 patients who had liver resection for HCC between 2012 and 2016, 147 met the inclusion criteria to act as the validation cohort. Patient demographics according to high and low LMR are shown in *Table* S1 (supporting information). A low LMR was associated with significantly lower OS (*P* = 0·032) and RFS (*P* = 0·005) rates than a high LMR (*Fig*. S5, supporting information).

IHC staining for PD‐L1 was conducted in 66 HCC samples, owing to a shortage of pathological sections. Twenty patients were positive for PD‐L1 at the tumour site. LMR was significantly lower in PD‐L1+ than in PD‐L1− patients (median (range) 4·71 (2·18–10·30) *versus* 4·15 (2·07–6·16) respectively; *P* = 0·045). However, there was no statistically significant association between PD‐L1 expression and NLR (*P* = 0·205) or PLR (*P* = 0·105).

### PD‐L1 expression in HCC cell lines

Expression of PD‐L1 protein was increased by IFN‐γ and co‐culture with THP‐1 to a level significantly higher than that in untreated control cells (*P* = 0·036) (*Fig*. S6, supporting information).

## Discussion

This study has shown that low LMR is an independent poor predictor of OS and RFS in patients with HCC. The results also suggest that patients with HCC and a low preoperative LMR have more aggressive tumour behaviour, including a larger tumour size and higher serum AFP concentration, than patients with a high preoperative LMR.

NLR, PLR and LMR are considered systemic inflammatory biomarkers and have been investigated in patients with various types of cancer^17^, ^18^, ^19^. High PLR was associated with worse OS and RFS in (mostly non‐cirrhotic) patients with HCC^20^. These findings were not validated by another study^21^, in which both NLR and LMR, but not PLR, were independently predictive of RFS in HCC associated with HBV‐related cirrhosis.

Some recent studies^9^, ^10^ have demonstrated that a lower LMR was associated with worse survival in patients with HCC. A lower LMR could result from a reduced level of lymphocytes, an increased level of monocytes, or both. However, the literature^9^, ^10^ lacks optimal cutoff values.

A significant relationship between low LMR and expression of PD‐L1 was found in the present study, whereas high LMR with PD‐L1− expression was a prognostic factor for improved OS and RFS.

Of interest, LMR had a statistically significant association with PD‐L1 expression compared with NLR and PLR in patients with HCC. Several reports^22^, ^23^, ^24^, ^25^ have suggested that PD‐L1 expression in tumour cells could increase in response to cytokine exposure from tumour‐associated immune cells (tumour‐infiltrating lymphocytes) in the tumour microenvironment^22^. In the present study, expression of PD‐L1 protein in HCC cells was increased by IFN‐γ or co‐culture with THP‐1 cells to a level significantly higher than that in untreated control cells. However, a low LMR might indicate increased levels of monocyte‐derived cells in the HCC microenvironment, which might result in increased PD‐L1 expression of HCC cells in response to cytokines from the immune cells.

Several clinical trials have evaluated various immune checkpoint inhibitors and combination therapy^23^, including anticytotoxic T lymphocyte‐associated antigen 4 antibody and PD‐1 antibody, which were reported to show promising outcomes^24^, ^25^.

This study is limited by its retrospective design and the suboptimal ROC curve AUC values. Multi‐institutional studies with greater numbers of patients and additional data would be required to confirm the present results.

## Supporting information


**Table S1** Characteristics of patients with hepatocellular carcinoma who underwent hepatic resection in the validation group
**Fig. S1** Receiver operating characteristic (ROC) curve using lymphocyte‐to‐monocyte ratio (LMR) as a predictor of overall survival after hepatic resection; cutoff value of 4·28. Area under the ROC curve (AUC) 0·616.
**Fig. S2 a** Overall and **b** recurrence‐free survival in patients with hepatocellular carcinoma with and without cirrhosis according to high or low lymphocyte‐to‐monocyte ratio (LMR).
**Fig. S3 a** Overall and **b** recurrence‐free survival in patients with hepatoculleular carcinoma with an without microvascular invasion according to high or low lymphocyte‐to‐monocyte ratio (LMR).
**Fig. S4 a** Overall and **b** recurrence‐free in patients with high or low lymphocyte‐to‐monocyte ratio (LMR) and programmed death ligand (PD‐L) 1‐positive and ‐negative expression: LMR high/PD‐L1 (−) (*n =* 134) (blue lines); LMR high/PD‐L1 (+) (*n* = 19) (green lines); LMR low/PD‐L1 (−), (*n* = 47) (yellow lines); LMR low/PD‐L1 (+) (*n* = 23) (red lines).
**Fig. S5** Overall and recurrence‐free survival after resection of hepatocellular carcinoma in patients with high or low lymphocyte‐to‐monocyte ratio (LMR) in the validation group.
**Fig. S6** Programmed death ligand (PD‐L) 1 expression in hepatocellular carcinoma cell lines. Cells were untreated (control) or treated with interferon (IFN) γ (100 ng/ml) or co‐cultured with THP‐1 cells for 48 h. PD‐L1 protein expression was evaluated by western blotting. Results are representative of three independent experiments. *P = 0·036 (treated or co‐cultured *versus* untreated control cells; Mann–Whitney *U* test).
