# Supplementary material for: Prognostic significance of inflammatory biomarkers in hepatocellular carcinoma following hepatic resection
Source: BJS Open. 2019 Apr 29;3(4):500–8. doi: 10.1002/bjs5.50170 (PMC6677099; doi:10.1002/bjs5.50170)
Supplement: Supplementary file 1 — Table S1 Characteristics of patients with hepatocellular carcinoma who underwent hepatic resection in the validation group Fig. S1 Receiver operating characteristic (ROC) curve using lymphocyte‐to‐monocyte ratio (LMR) as a predictor of overall survival after hepatic resection; cutoff value of 4·28. Area under the ROC curve (AUC) 0·616. Fig. S2 a Overall and b recurrence‐free survival in patients with hepatocellular carcinoma with and without cirrhosis according to high or low lymphocyte‐to‐monocyte ratio (LMR). Fig. S3 a Overall and b recurrence‐free survival in patients with hepatoculleular carcinoma with an without microvascular invasion according to high or low lymphocyte‐to‐monocyte ratio (LMR). Fig. S4 a Overall and b recurrence‐free in patients with high or low lymphocyte‐to‐monocyte ratio (LMR) and programmed death ligand (PD‐L) 1‐positive and ‐negative expression: LMR high/PD‐L1 (−) (n = 134) (blue lines); LMR high/PD‐L1 (+) (n = 19) (green lines); LMR low/PD‐L1 (−), (n = 47) (yellow lines); LMR low/PD‐L1 (+) (n = 23) (red lines). Fig. S5 Overall and recurrence‐free survival after resection of hepatocellular carcinoma in patients with high or low lymphocyte‐to‐monocyte ratio (LMR) in the validation group. Fig. S6 Programmed death ligand (PD‐L) 1 expression in hepatocellular carcinoma cell lines. Cells were untreated (control) or treated with interferon (IFN) γ (100 ng/ml) or co‐cultured with THP‐1 cells for 48 h. PD‐L1 protein expression was evaluated by western blotting. Results are representative of three independent experiments. *P = 0·036 (treated or co‐cultured versus untreated control cells; Mann–Whitney U test). [file BJS5-3-500-s001.docx]

**BJS5_50170**

**Prognostic significance of inflammatory biomarkers in hepatocellular carcinoma following hepatic resection**

**S. Itoh, K. Yugawa, M. Shimokawa, S. Yoshiya, Y. Mano, K. Takeishi, T. Toshima, Y. Maehara, M. Mori and T. Yoshizumi**

**Table S1** Characteristics of patients with hepatocellular carcinoma who underwent hepatic resection in the validation group

| Variable | High LMR (n=85) | Low LMR (n = 62) | *p*-value |
| --- | --- | --- | --- |
| Age (years) | 70 (34-86) | 69 (39-87) | 0.791 |
| Sex, male/female | 60/25 | 53/9 | 0.047 |
| BMI (kg/m^2^) | 23.3 (17.6-33.3) | 22.4 (14.7-35.9) | 0.101 |
| HBs-Ag positive | 13 (15.2%) | 7 (11.2%) | 0.484 |
| HCV-Ab positive | 34 (40.0%) | 24 (38.7%) | 0.874 |
| Diabetes mellitus | 25 (29.4%) | 25 (40.9%) | 0.146 |
| Child Pugh A/B | 85/0 | 60/2 | 0.176 |
| Oesophageal varix | 3 (3.5%) | 4 (6.4%) | 0.745 |
| Total bilirubin (mg/dl) | 0.7 (0.2-2.1) | 0.7 (0.3-1.4) | 0.926 |
| Albumin (g/dl) | 4.1 (3.2-4.8) | 4.0 (2.7-5.1) | 0.099 |
| Prothrombin time (%) | 94 (71-123) | 92 (66-121) | 0.379 |
| ICGR15 (%) | 10.3 (0.1-71.6) | 10.4 (1.1-36.9) | 0.819 |
| AFP (ng/ml) | 7.9 (1.2-50649) | 25 (2.1-165774) | 0.048 |
| DCP (mAU/ml) | 53 (2-63582) | 221 (12-175610) | 0.001 |
| Tumor size (cm) | 3.0 (1.0-17) | 4.1 (1.0-20) | 0.024 |
| Solitary/Multiple | 69/16 | 46/16 | 0.311 |
| Poor differentiation | 22 (25.8%) | 25 (40.9%) | 0.054 |
| Microvascular invasion | 12 (14.1%) | 21 (33.8%) | 0.004 |
| Microscopic intrahepatic metastases | 10 (11.7%) | 19 (30.6%) | 0.004 |
| Liver cirrhosis | 5 (5.8%) | 7 (11.2%) | 0.237 |
| Operation time (min) | 283 (65-845) | 284 (96-659) | 0.898 |
| Blood loss (ml) | 300 (0-4051) | 485 (16-5391) | 0.074 |
| Blood transfusion | 11 (12.9%) | 10 (16.1%) | 0.585 |

Data are presented as n (%) or median (range). LMR, lymphocyte-to-monocyte ratio; BMI, body mass index; HBs-Ag, hepatitis B surface antigen; HCV-Ab, hepatitis C virus antibody; ICGR15, indocyanine green retention rate at 15 min; AFP, alpha-fetoprotein; DCP, *des*-gamma-carboxyprothrombin.

**Fig. S1** Receiver operating characteristic (ROC) curve using lymphocyte-to-monocyte ratio (LMR) as a predictor of overall survival after hepatic resection; cutoff value of 4.28. Area under the ROC curve (AUC) 0.616.

**Fig. S2 a** Overall and **b** recurrence-free survival in patients with hepatocellular carcinoma with and without cirrhosis according to high or low lymphocyte-to-monocyte ratio (LMR).

**Fig. S3 a** Overall and **b** recurrence-free survival in patients with hepatoculleular carcinoma with an without microvascular invasion according to high or low lymphocyte-to-monocyte ratio (LMR).

**Fig. S4 a** Overall and **b** recurrence-free in patients with high or low lymphocyte-to-monocyte ratio (LMR) and programmed death ligand (PD-L) 1-positive and -negative expression: LMR high/PD-L1 (−) (*n =*134) (blue lines); LMR high/PD-L1 (+) (*n* = 19) (green lines); LMR low/PD-L1 (−), (*n* =47) (yellow lines); LMR low/PD-L1 (+) (*n* = 23) (red lines).

**Fig. S5** Overall and recurrence-free survival after resection of hepatocellular carcinoma in patients with high or low lymphocyte-to-monocyte ratio (LMR) in the validation group.

**Fig. S6** Programmed death ligand (PD-L) 1 expression in hepatocellular carcinoma cell lines. Cells were untreated (control) or treated with interferon (IFN) γ (100 ng/ml) or co-cultured with THP-1 cells for 48 h. PD-L1 protein expression was evaluated by western blotting. Results are representative of three independent experiments. **P* = 0.036 (treated or co-cultured *versus* untreated control cells; Mann–Whitney *U* test).
